# Healthberry 865^®^ and a Subset of Its Single Anthocyanins Attenuate Oxidative Stress in Human Endothelial In Vitro Models

**DOI:** 10.3390/nu14142917

**Published:** 2022-07-16

**Authors:** Philipp Ockermann, Rosario Lizio, Jan Hansmann

**Affiliations:** 1 Institute for Tissue Engineering and Regenerative Medicine, University Hospital Wuerzburg, Roentgenring 11, 97070 Wuerzburg, Germany; jan.hansmann@uni-wuerzburg.de; 2 Evonik Operations GmbH, 63457 Hanau, Germany; rosario.lizio@evonik.com

**Keywords:** anthocyanins, reactive oxygen species, HUVEC, microvascular endothelial cells

## Abstract

Oxidative stress and inflammation play a pivotal role in the development of cardiovascular diseases, an ever-growing worldwide problem. As a non-pharmacological approach, diet, especially a flavonoid-rich diet, showed promising results in the reduction of cardiovascular diseases and alleviation of their symptoms. In this study, in vitro systems based on human microvascular endothelial cells (hmvEC) and human umbilical cord endothelial cells (HUVEC) were established to determine the effect of Healthberry 865^®^ (HB) and ten of its relating single anthocyanins on oxidative stress. Furthermore, five metabolites were used in order to examine the effect of anthocyanin’s most common breakdown molecules. The results showed an effect of HB in both models after 24 h, as well as most of its single anthocyanins. Cyanidin-rutinoside, peonidin-galactoside, and petunidin-glucoside had a model-specific effect. For the metabolites, phloroglucinaldeyhde (PGA) showed an effect in both models, while vanillic acid (VA) only had an effect in HUVEC. When combined, a combination of several anthocyanins did not have a cumulative effect, except for combining glucosides in hmvEC. The combination of PGA and VA even revealed an inhibitive behavior. Overall, the study demonstrates the antioxidative effect of HB and several of its single anthocyanins and metabolites, which are partially model specific, and coincides with animal studies.

## 1. Introduction

Anthocyanins are a widely-distributed subgroup of flavonoids, a class of secondary plant metabolites responsible for pigmentation, UV-protection, and other protective functions [1,2]. Depending on the pH, they appear red, purple, or blue. They can be further divided based on their substitutions at the flavylium cation and their sugar moieties. Natural dietary sources are dark and red, such as berries as well as vegetables [3]. Daily anthocyanins intake is estimated to range between 12.5 mg/d and 65 mg/d in the U.S. and Europe [4,5]. Currently, they are an upcoming topic as nutraceuticals and as experimental approaches that suggest that intake decreases oxidative stress, blood pressure, cholesterol, and inflammation markers, thus resulting in vaso- and neuroprotection [6,7,8].

Typically, the bioavailability of anthocyanins in blood plasma was considered to be as low as 0.1% [9,10]. In contrast, ^13^C isotopically-labeled cyanidin-glucoside showed a relative bioavailability of ~12%, revealing a serum concentration in the low micromolar range [11,12]. Additionally, labeled metabolites were found that showed a maximum concentration at a later time point than the parent anthocyanins [11,12].

Anthocyanins and their metabolites have been extensively studied for their antioxidant activity. While reactive oxygen species (ROS) are produced and needed in cellular compartments, an excess can have detrimental effects, especially in blood vessels [13]. A decrease in angiotensin-II-stimulated superoxide production was found for vanillic acid and proto-catechuic acid in human cell cultures [14], while a significant reduction in ROS levels was found using malvidin-3-glucoside and -galactoside [15]. Moreover, the production of xanthine oxidase-1 (XO-1) was reduced. Simultaneously, the release of superoxide dismutase (SOD) and heme oxygenase-1 (HO-1) was strengthened. Similar results were obtained by others [16,17]. Supplementation of isolated anthocyanins also resulted in the short-term improvement of serum antioxidant capacity [18], therefore decreasing oxidative stress and potentially the risk of related diseases [19]. Black currant and pomegranate juice, both containing high amounts of anthocyanins, have been shown to improve the serum antioxidative status in vivo [20].

Due to the positive effect on health, anthocyanin-rich extracts, often from black currant and pomegranate, became the focus in the development of food supplements. The effect of Healthberry 865^®^ (HB), a standardized extract with defined amounts of anthocyanins from bilberries (*Vaccinium myrtillus*) and black currant (*Ribes nigrum*), and its single anthocyanins, have already been analyzed in mice arteries in an ex vivo setup [21]. HB resulted in the reduction of oxidative stress, which was mediated by specific anthocyanins.

The aim of the study was to better understand HB’s effects in humans. With ex vivo experiments being complex, time-consuming, and comparatively challenging, and animal studies in general being difficult to convert into human terms, the aim was to create a human test system that is simple to use but complex enough to yield significant results.

This study investigated HB regarding its effect on antioxidative potential, as well as the effect of ten of its single anthocyanins in two different human endothelial cell models at a realistic concentration range. The results were subsequently compared with the already-existing data to validate the system. To go one step further, it was investigated whether the same or similar effects can be evoked by anthocyanins’ metabolites by using five phase-I and -II metabolites. Additionally, the cumulative effects of these anthocyanins and metabolites showing the strongest antioxidative potential were tested.

## 2. Materials and Methods

### 2.1. Chemicals and Reagents

Medox/Healthberry and the single anthocyanins were provided by Evonik. Dulbecco′s Phosphate Buffered Saline without calcium chloride and magnesium chloride (PBS-, D8537), PBS+ (D8662) and 2′,7′-Dichlorofluorescin diacetate (DCF-DA, D6883), vanillic acid (VA, 94770), hippuric acid (HA, 11200), protocatechuic acid (PCA, 37580), phloroglucin aldehyde (PGA, T65404), and ferulic acid (FA, W518301) were obtained from Sigma Aldrich (St. Louis, MI, USA). Furthermore, Hanks’ Balanced Salt Solution (HBSS, 14025-92) was obtained from Gibco (Walham, MA, USA), VascuLife EnGS-Mv (LS-1035) was obtained from LifeLine (Singapore), and Fetal Calf Serum (FCS, P30-3033) was obtained from PAN Biotech (Aidenbach, Germany). The anthocyanins and metabolites were stored dry at −20 °C and dissolved in DMSO before using. The final concentration of DMSO in the medium was 0.1% and the controls were used with 0.1% DMSO only.

### 2.2. Cell Culture and Treatment

Human microvascular endothelial cells (hmvEC) have been isolated from the dermis of foreskin biopsies, as had been described elsewhere [22] according to the ethical approval granted by the Institutional Ethics Committee of the University Hospital Würzburg (approval number 182/10), and pooled from three different donors. Briefly, endothelial cells were isolated from the dermis of foreskin biopsies and their lineage morphology was confirmed by their cobblestone pattern. If fibroblasts were found, the cells were sorted by using magnetic-associated cell sorting beasts directed against CD31 as endothelial markers (Miltenyi). Human umbilical vein endothelial cells (HUVEC) were obtained from Cellsystems (FC-0003). HUVECS were used in passage 5 and hmvEC in passage 2. The cells were cultured in T150 flasks (TPP, 90151) in VascuLife EnGS-Mv medium at 37 °C, 95% humidity, 5% CO_2_, and the medium was changed every two to three days.

For the experiment, cells were seeded in 96-well black flat bottom plates at 10 × 10^3^ cells on the plate ground and grown until confluence in VascuLife EnGS-Mv medium at 37 °C, 95% humidity, and 5% CO_2_. They were further silenced with culture medium without FSC for 6 h and then incubated with the respective anthocyanin concentration in culture medium without FCS for 24 h.

### 2.3. Cytotoxicity Test

To exclude the cytotoxicity of the substances, cell viability was examined with MTT and CellTiterGlo (Promega, G7571), using the highest concentrations (100 µg/mL, respectively 200 µM). The vehicle and normal medium served as controls. Cells were incubated with the respective substance and subsequently tested. For the MTT test, cells were incubated in medium with 1 mg/mL MTT for 4 h, afterwards, the medium was removed, and the formazan was extracted with DMSO. The absorption was measured at 570 nm with a TECAN Infinite 200 Microplate reader. For CellTiterGlo, cells were washed with PBS+ and CellTiterGlo reagent and culture medium was added. After 2 min shaking and 10 min further incubation, luminescence was measured with a TECAN Infinite 200 Microplate reader.

### 2.4. Analysis of Total ROS Production

Cells were incubated with 10 µM DCF-DA in FCS-free medium for 30 min at 37 °C, and subsequently washed two times with HBSS and then left for an additional 30 min at 37 °C in HBSS for oxidation of the dye. Fluorescence measurements took place with a TECAN Infinite 200 Microplate reader at 492 and 527 nm excitation/emission with several single measurement points inside of the well to achieve a mean fluorescence value from the whole plate ground. 

### 2.5. Statistical Analysis 

The results are expressed as mean ± SD. The statistical analysis was done with IBM SPSS Statistics (v26). A one-way ANOVA was performed with subsequent Tukey-HSD and Bonferroni as post-hoc tests.

## 3. Results

A viability test of all anthocyanins and metabolites were found to be non-cytotoxic at a concentration of 100 µg/mL or 200 µM, respectively, with the normal medium and vehicle serving as controls (Figure S1).

**Figure 1:**
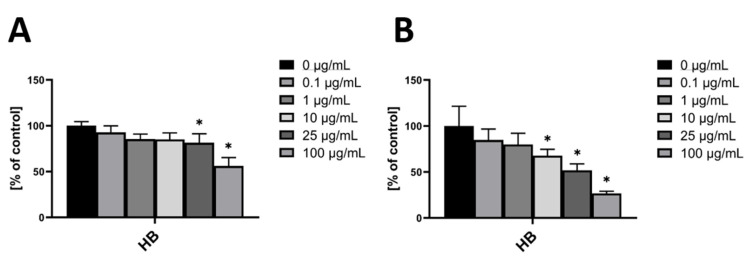
DCF-DA fluorescence relative to control with the respective concentrations of HB in HUVEC (**A**) and hmvEC (**B**); results are expressed as mean ± SD; * *p* < 0.05 compared to 0 µg/mL HB; *n* = 4 technical replicates.

In HUVEC, incubation with HB for 24 h resulted in a significant decrease of ROS at concentrations of 25 µg/mL and 100 µg/mL, when compared to vehicle control (Figure 1A). In hmvEC, incubation with HB for 24 h resulted in a significant decrease of ROS at concentrations of 10 µg/mL and higher (Figure 1B). As HB is a mixture of different anthocyanins, further investigation on the effects of its single anthocyanins was needed.

**Figure 2:**
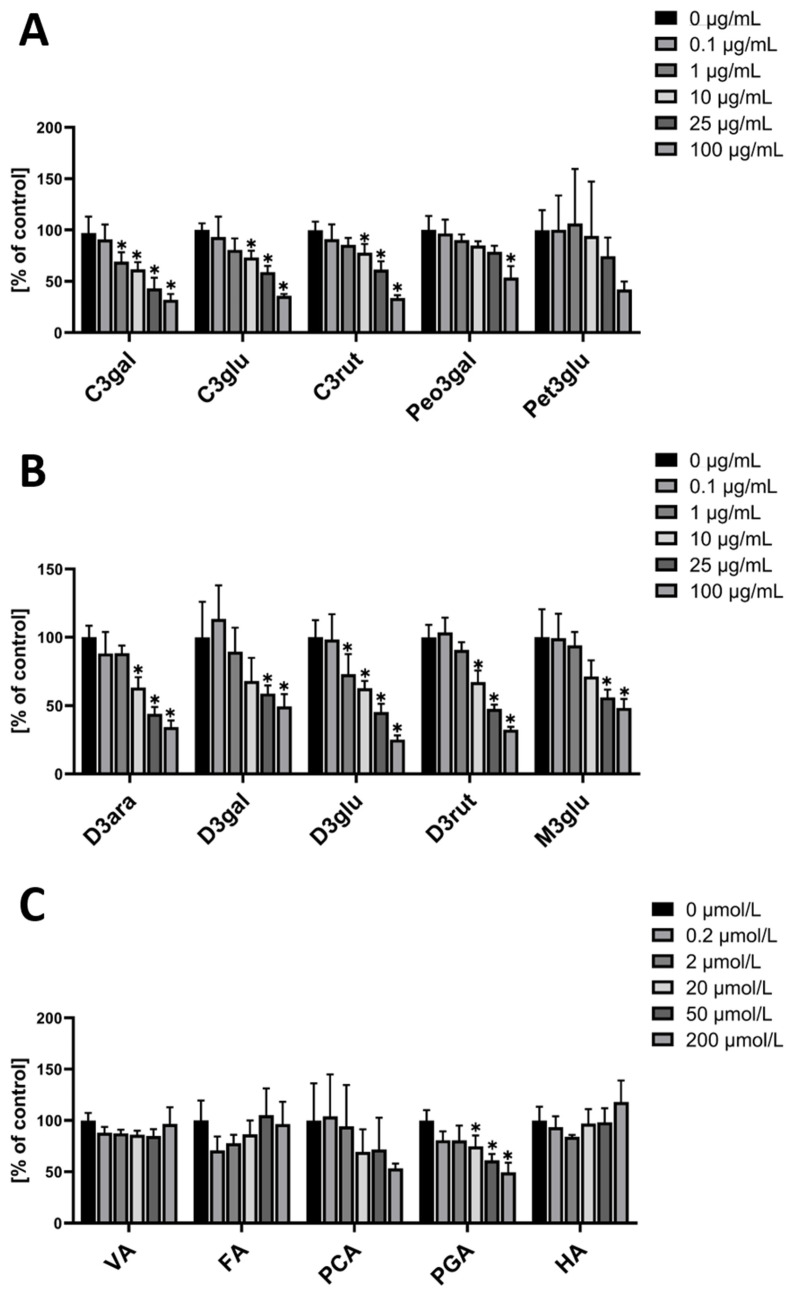
DCF-DA fluorescence relative to control in hmvEC with the respective concentrations of single anthocyanins (**A**,**B**) and the respective concentrations of anthocyanin metabolites (**C**); results are expressed as mean ± SD; * *p* < 0.05 compared to 0 µg/mL of the respective substance; *n* = 4 technical replicates.

When hmvEC were incubated with single anthocyanins, the used cyanidins (-galactoside (C3gal), -glucoside (C3glu), and –rutinoside (C3rut)) all resulted in a significant decrease at concentrations of 10 µg/mL and higher (Figure 2A). When using C3gal, a significant decrease was even observed at 1 µg/mL. Generally, they all show a similar pattern with a concentration-dependent decrease. Peonidin-3-galactoside (Peo3gal) showed a different result, with only the highest concentration being significantly decreased, while the other concentrations presented a plateau. For petunidin-3-galactoside (Pet3glu), there was no significant effect from any of the concentrations. While the highest concentration visibly seemed to be decreased, it failed the statistical significance test.

For the delphinidins (-arabinoside (D3ara), -galactoside (D3gal), -glucoside (D3glu), and –rutinoside (D3rut)), a similar result as for the cyanidins was found. Except for D3gal, all tested delphinidins resulted in a significant ROS decrease at concentrations of 10 µg/mL and higher (Figure 2B). D3gal only showed a significant effect at the two highest concentrations, while D3glu even showed a significant decrease at 1 µg/mL. Malvidin-3-glucoside (M3glu) had a significant ROS decreasing effect at the two highest concentrations, with a plateau at the two lowest concentrations.

As anthocyanins are rapidly metabolized in the human body, it was of interest to also test the main metabolites for their respective antioxidative effect. One of the five tested had a significant effect. PGA significantly decreased ROS at a concentration of 20 µM and higher. (Figure 2C)

**Figure 3:**
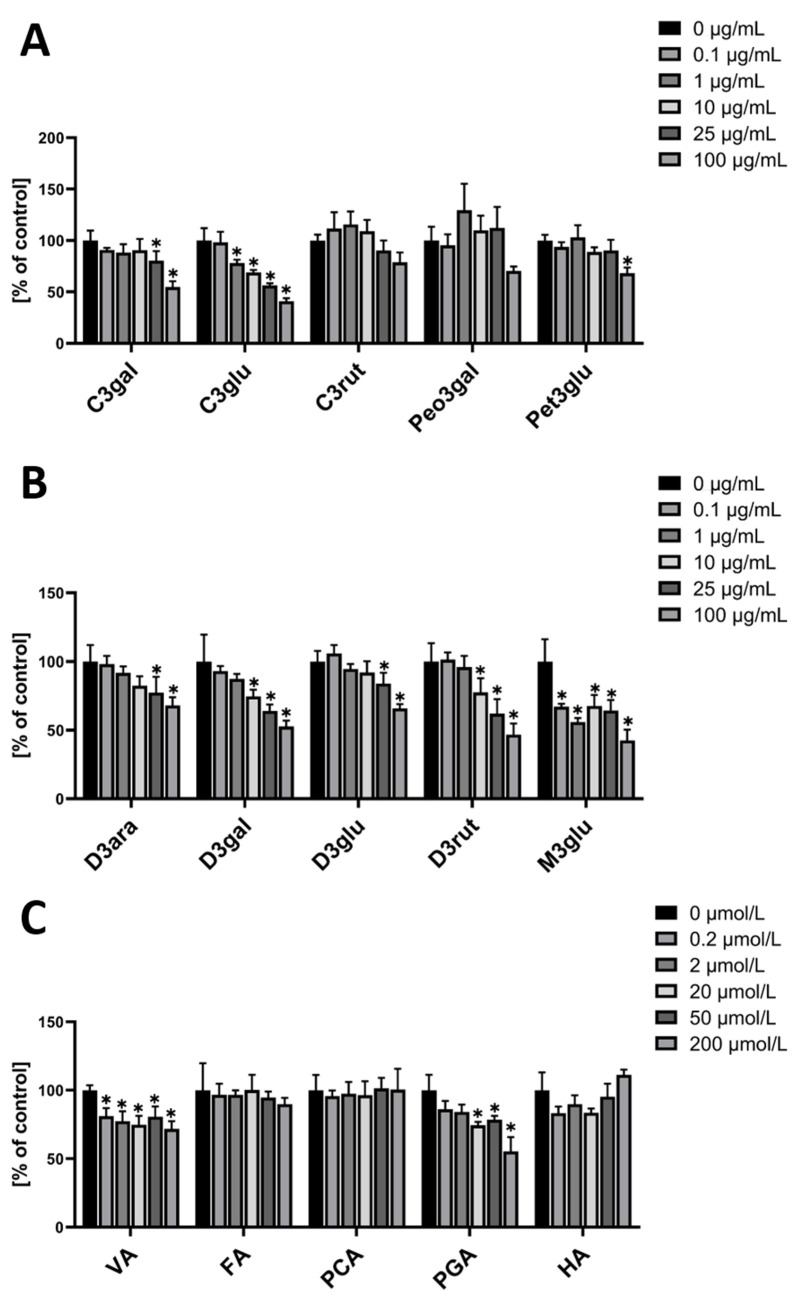
DCF-DA fluorescence relative to control in HUVEC with the respective concentrations of single anthocyanins (**A**,**B**) and the respective concentrations of anthocyanin metabolites (**C**); results are expressed as mean ± SD; * *p* < 0.05 compared to 0 µg/mL of the respective substance; *n* = 4 technical replicates.

In contrast to hmvEC, the cyanidins had different effects in HUVEC. While C3rut did not have a significant effect on ROS production at any of the used concentrations, C3glu had a concentration-dependent effect from 1 µg/mL and higher. C3gal, however, showed an effect at the two highest concentrations, while having a plateau at lower concentrations. Peo3gal did not have any significant effect, while Pet3glu only had an effect at the highest concentration (Figure 3A).

In the group of delphinidins, all four showed a significant ROS decreasing effect at the two highest concentrations. D3rut and D3gal additionally showed an effect at 10 µg/mL (Figure 3B). M3glu is the only anthocyanin that had a significant effect at a concentration of 0.1 µg/mL, however, at higher concentrations, the effect did not increase but stagnated on a plateau. Of all the metabolites, VA and PGA were able to evoke a significant ROS decreasing effect, while the others did not have any significance. VA’s effect was visible from a concentration of 0.2 µM onwards, while PGA’s effect started to be significant from 20 µM on (Figure 3C).

Overall, the tested anthocyanins seem to have a less strong effect in HUVEC than they have in hmvEC. Still, there is a concentration dependent effect with some of the anthocyanins. In the case of the metabolites, PGA showed an effect in HUVEC as well as in hmvEC. Additionally, VA also decreased ROS significantly in HUVEC, which does not appear to be dose-dependent but was already significant at the lowest concentration.

Since HB itself, as a mixture of several anthocyanins, and various single anthocyanins showed an effect in both cell types, the next step was to combine specific anthocyanins. This was done based on the previous results. The following anthocyanin combinations were used (Table 1).

The rationale behind these mixtures was that Mix 1 groups all cyanidins, as they showed an effect in both cell types, at least in the two highest concentrations—except for C3rut, which was only significant in hmvEC. Mix 2 groups all delphinidins, as they showed an effect in both cell types, at least in the two highest concentrations. Mix 3 groups all glucosides, as all of them showed an effect in both cell types. M3glu even showed an effect at the lowest concentration in HUVEC. Mix 4 groups all rutinosides, as, except for C3rut in HUVEC, they all showed an effect in both cell types. Mix 5 is a mixture of all anthocyanins that were used in the other combinations. Mix 6 groups the two metabolites that showed an effect.

**Figure 4:**
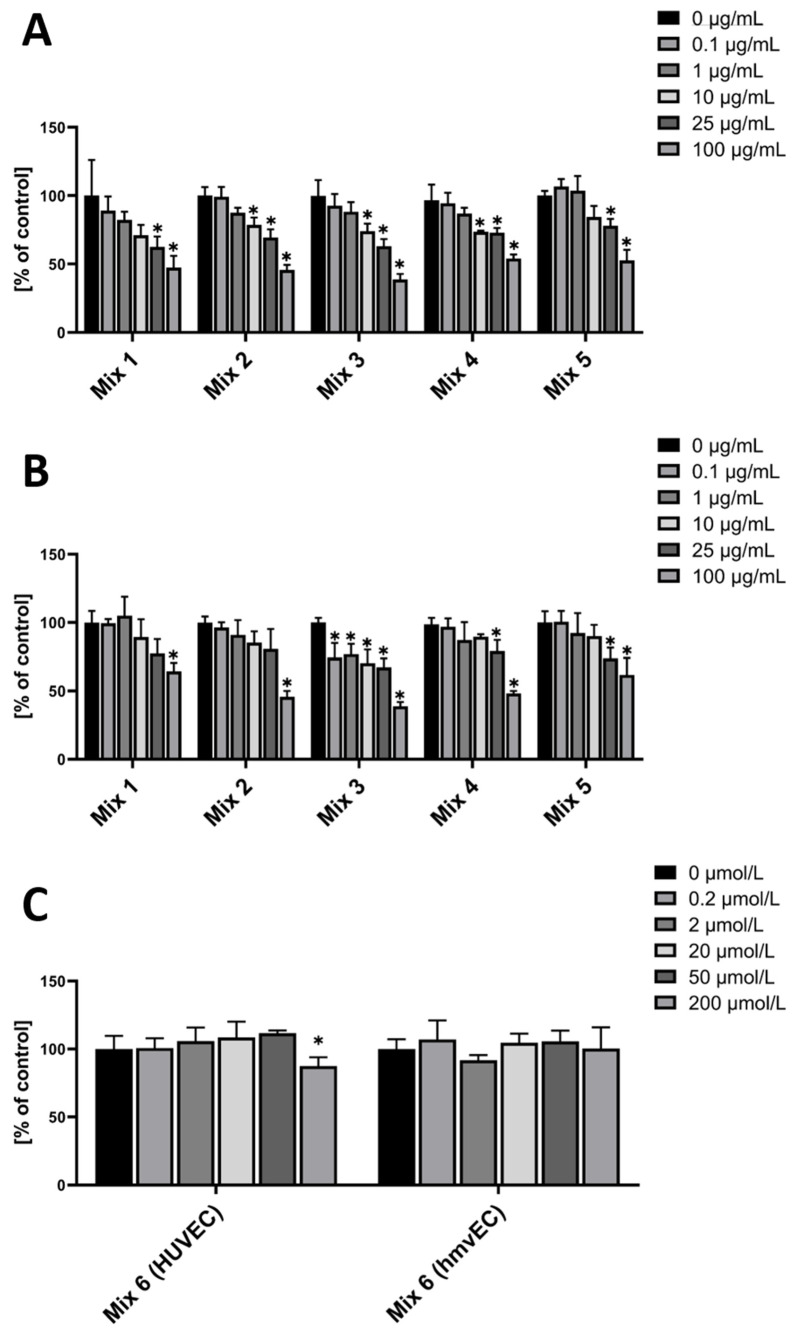
DCF-DA fluorescence relative to control in HUVEC (**A**) and hmvEC (**B**) with the respective cumulative concentration and respective anthocyanin or metabolite (**C**) combinations; results are expressed as mean ± SD; * *p* < 0.05 compared to 0 µg/mL of the respective mix; *n* = 4 technical replicates.

In HUVEC, Mixes 1 and 5 showed a similar pattern, all decreasing ROS level in the two highest concentrations, with Mixes 2 and 4 even at 10 µg/mL (Figure 4A).

In hmvEC, Mix1, comprised of the three tested cyanidins, only had a significant effect at the highest concentrations, whereas the single anthocyanins decreased ROS levels further at lower concentrations. The same was found for Mix 2 in hmvEC, which was comprised of all four tested delphinidins, and Mix 4 (all tested galactosides), and Mix 5. Mix 3, which was comprised of the three tested glucosides, however, did show a significant effect even at the lowest concentrations, plateauing then except for the highest concentration (Figure 4B).

For Mix 6, the combination of the two metabolites that did have a significant effect in the first experiment, there was only a significant effect in HUVEC and only at the highest concentrations. While both VA and PGA had an effect in HUVEC at 0.2 µM and 20 µM, respectively, the combination did not compete on this level (Figure 4C).

## 4. Discussion

Reactive oxygen species play a major role in the development of endothelial dysfunction. The ability of anthocyanins and anthocyanidins to decrease ROS in general has been shown in several studies already [15,16,17,18,23,24]. However, only few different anthocyanins were used, probably due to availability, with a main focus on cyanidin-glucoside. Also, umbilical cord endothelial cells are most commonly used for cell models. Here, an antioxidant effect of a blueberry and black currant extract (Healthberry-865^®^, HB), mainly comprised of anthocyanins and multiples of them, could be shown in two different cell models. For this, not only were umbilical cord cells, but also microvascular endothelial cells from foreskin, used; the first being easily accessible, widely used, and an accepted cell model for blood vessels, and the latter being a rather uncommon cell model representing smaller vessels. The tested concentrations were chosen to represent physiological relevant concentrations, as well as higher concentrations used in most publications. Oxidative stress was indirectly induced by using culture medium without FCS, which increased ROS after the initial starving phase (Figure S2)—however, not being cytotoxic. While all tested delphinidins, C3gal, C3glu, and M3glu, showed a similar effect in both cell models, the other ones showed a model-specific behavior. While Pet3glu did not have a significant effect in hmvEC, it did so in HUVEC, although only at the highest concentration. In contrast, C3rut and Peo3gal did not effect HUVEC, but did affect hmvEC. It therefore seems that there is a cell- or model-specific effect of specific anthocyanins. Additionally, the effect of HB seems to be evoked by a specific subset of anthocyanins. Similar results were found by others [21] further strengthening the results from this model. HB is comprised of additional anthocyanins, but, unfortunately, they were not commercially available or were found impure after purchase. However, the most abundant anthocyanins in HB were studied and the results can therefore already give a decent overview of the subset specificity.

As anthocyanins are quickly metabolized after digestion (i.e., by intestinal microbiota [25,26]), their vasoprotective effect found in vivo might be mediated by metabolites [23]. These metabolites, i.e., vanillic acid or phloroglucinaldehyde, have been studied regarding their effect on inflammation [27,28]. Bharat et al. studied the effects of a blueberry metabolite mix, including hippuric acid and vanillic acid derivates, and found an amelioration of palmitate-induced ROS increase [23]. Others found vanillic acids to have an ameliorative effect when cells were stimulated with different ROS triggers [14,29]. Ho et al. found a protocatechuic acid and phloroglucinaldehyde to have a moderate inhibitory effect on xanthin oxidase (XO), a superoxide-producing enzyme [30]. When testing the effects of anthocyanin metabolites in this study, phloroglucinaldehyde was found to have an ameliorating effect on ROS production in both HUVEC and hmvEC. In HUVEC, vanillic acid had an effect at the lowest concentration already, but not in hmvEC. With this, it could not only be shown that anthocyanin metabolites in general have a similar effect as their parent anthocyanins, but also that there is a model-specific effect of vanillic acid. This could be of interest for future studies or for nutritional supplements, as, if metabolites evoke the same effect as anthocyanins, they might be used directly. In general, they are easier, more available, and cheaper than single anthocyanins, which are difficult to extract in a pure form.

While anthocyanin sources in nature (i.e., berries) contain not one but several different anthocyanins, the results from single anthocyanin studies are difficult to extrapolate for a whole source. For HB, the anthocyanin composition, but not the individual percentage, is known. Therefore, we decided not to use its ratio, but to create combinations with the most powerful anthocyanins from the previous experiments to examine potential cumulative effects, which could be obscured in the whole source by low proportions.

In hmvEC, the combination of anthocyanins did not resemble their effects when individually used. The general effect was weaker and only occurred at higher concentrations. This is insofar expectable, as the concentration is the combination of all used anthocyanins in the mix, while the individual concentration is only a part, depending on the mix either being a ½, ⅓, ¼, or ⅛. The only exception was Mix 3, the combination of C3glu, D3glu, and M3glu. It seems that combining glucosides has a cumulative effect to some degree, while combining rutinosides, as well as cyanidins and delphinidins in general, does not. This effect does resemble the one found in Ref. [31].

In HUVEC, all mixtures show a similar pattern that resembles the pattern in the single anthocyanin experiment. As the individual anthocyanins are only a fraction of the total concentration, a cumulative effect of the respective concentrations can be shown here. However, the effect is not stronger than the effect of the strongest individual anthocyanin. Overall, it could be shown that there is a cell- or model-specific effect of anthocyanin mixtures, which is in line with our previous findings when testing single anthocyanins.

When the two metabolites were combined that did show an effect in HUVEC, as well as in hmvEC—namely vanillic acid and phloroglucinaldehyde—the effect was small. Only the highest concentration used, and only in HUVEC, were able to provide a significant effect. This is insofar surprising, as VA showed an effect in HUVEC at 0.2 µM already. The results here indicate that there is an inhibitory effect when combining these two metabolites, which has not been shown before.

Overall, the models used in this study were able to reproduce results found by others (e.g., Ref. [21]) in more complex systems while being comparatively simple. This makes it a scalable testing system, suitable for further studies with high throughput.

## 5. Conclusion

Overall, it could be shown that the blueberry and black currant extract HB attenuated oxidative stress in both, HUVEC and hmvEC. Its single components C3gal, C3glu, M3glu, all used delphinidins, and the anthocyanin metabolite PGA also showed an effect in both models. C3rut and Peo3gal affected only hmvECs, while Pet3glu and the metabolite VA only affected HUVECs, indicating a cell- or model-specific effect. When combining several single anthocyanins in hmvEC, only a combination of glucosides showed a weak cumulative effect, while the combination of rutinosides, cyanidins, or Delphinidins did not. In HUVEC, no cumulative effect could be found. Combining PGA and VA did only have an effect in HUVEC in the highest concentration, indicating an inhibitory effect of these two metabolites. In general, the models could reproduce results found by others in ex vivo systems, while being scalable for higher throughput studies. 

## Figures and Tables

**Table 1:** Anthocyanin mixtures used; concentrations were equimolar cumulative.

Name	Anthocyanins
Mix 1	C3gal + C3glu + C3 rut
Mix 2	D3ara + D3gal + D3glu + D3rut
Mix 3	C3glu + D3glu + M3glu
Mix 4	C3rut + D3rut
Mix 5	C3gal + C3glu + C3rut + D3ara + D3gal + D3glu + D3rut + M3glu
Mix 6	vanillic acid + phloroglucinaldehyde

## Data Availability

The data presented in this study are available on request from the corresponding author.

## Supplementary Materials

The following are available online at https://www.mdpi.com/article/10.3390/nu14142917/s1, Figure S1: Cell viability compared to control measured with MTT and CellTiterGlo of HUVEC (A+C) and hmvEC (B+D) after incubation with 100 µg/mL of the anthocyanins and 200 µM of the metabolites for 24 h in FCS-free culture medium; DMSO served as vehicle control, *n* = 3 technical replicates; Figure S2: Comparison of DCF-DA fluorescence in FCS-free medium relative to normal medium in HUVEC and hmvEC after 6 h incubation; * *p* < 0.05; *n* = 4 technical replicates.
