# Supplementary material for: Healthberry 865® and a Subset of Its Single Anthocyanins Attenuate Oxidative Stress in Human Endothelial In Vitro Models
Source: Nutrients. 2022 Jul 16;14(14):2917. doi: 10.3390/nu14142917 (PMC9325219; doi:10.3390/nu14142917)
Supplement: Supplementary file 1 [file nutrients-14-02917-s001.zip › nutrients-1801156-SI.pdf]

nutrients

MDPI

Supplementary Material

Healthberry 865® and a Subset of Its Single Anthocyanins Attenuate Oxidative Stress in Human Endothelial In Vitro Models

Philipp Ockermann [1,*], Rosario Lizio [2] and Jan Hansmann [1]

[1] Institute for Tissue Engineering and Regenerative Medicine, University Hospital Wuerzburg, Roentgenring 11, 97070 Wuerzburg, Germany; jan.hansmann@uni-wuerzburg.de
[2] Evonik Operations GmbH, 63457 Hanau, Germany; rosario.lizio@evonik.com
* Correspondence: philipp.ockermann@uni-wuerzburg.de

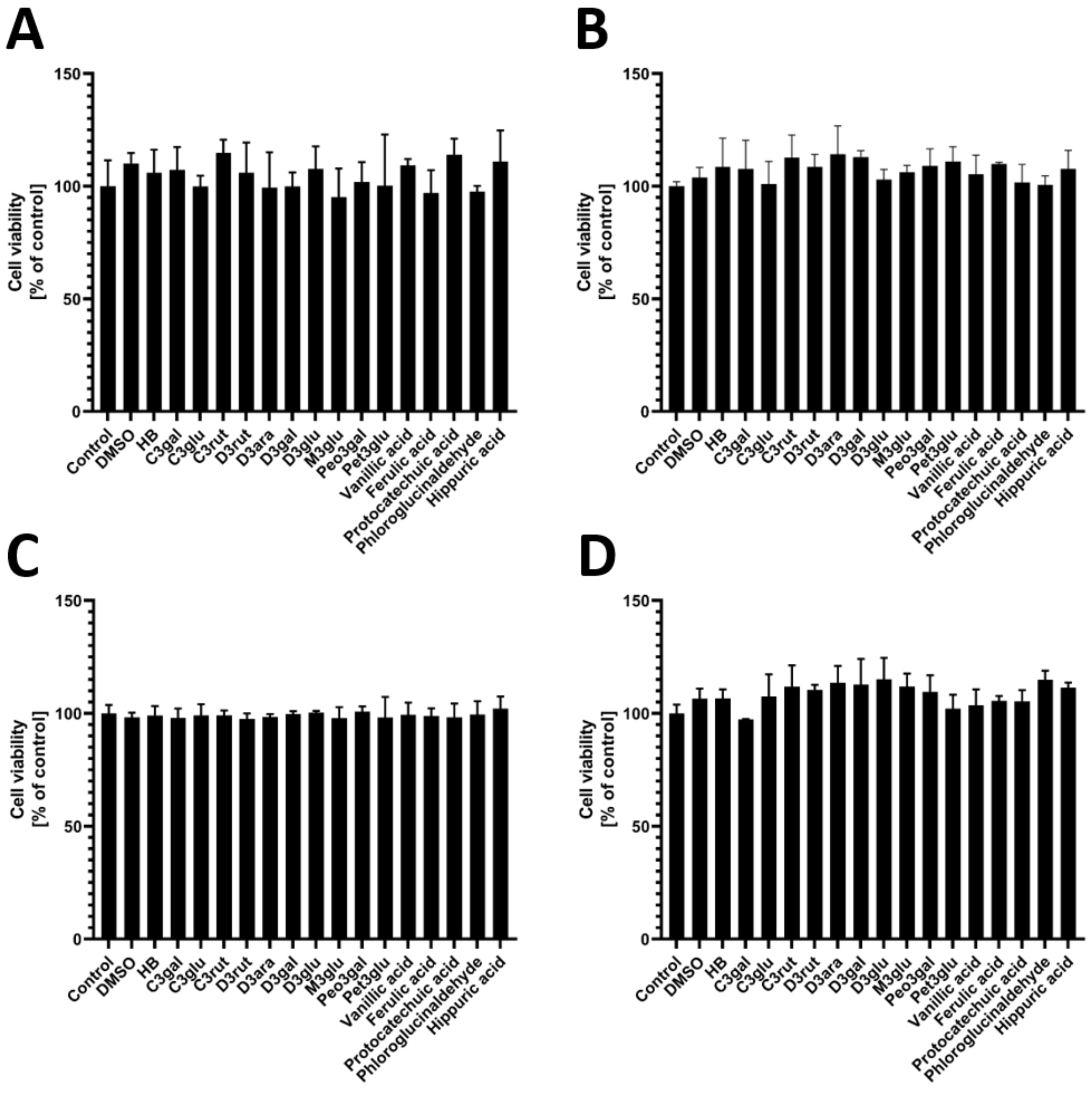

Figure S1: Cell viability compared to control measured with MTT and CellTiterGlo of HUVEC (A+C) and hmvEC (B+D) after incubation with 100 µg/mL of the anthocyanins and 200 µM of the metabolites for 24 h in FCS-free culture medium; DMSO served as vehicle control, n=3 technical replicates.

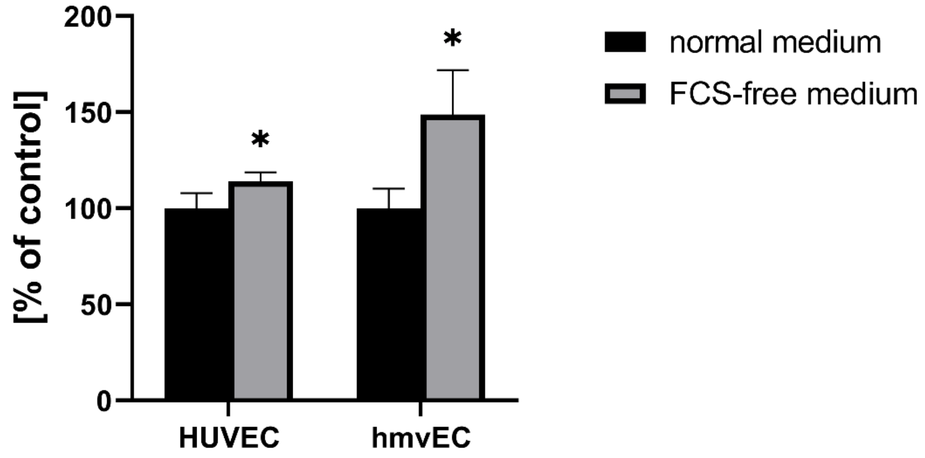

Figure S2: Comparison of DCF-DA fluorescence in FCS-free medium relative to normal medium in HUVEC and hmvEC after 6 h incubation; * $p < 0.05$; *n*=4 technical replicates.
